# Economic evaluation of management strategies for complex regional pain syndrome (CRPS)

**DOI:** 10.3389/fphar.2024.1297927

**Published:** 2024-01-22

**Authors:** Xiaofeng Zhou, Yi Zhou, Xiaofei Zhang, Feng Jiang

**Affiliations:** ^1^ Department of Pain, Taihe Hospital, Hubei University of Medicine, Shiyan, China; ^2^ Department of Neurosurgery, Taihe Hospital, Hubei University of Medicine, Shiyan, China; ^3^ Department of Neuro-Critical Care Medicine, Taihe Hospital, Hubei University of Medicine, Shiyan, China; ^4^ Department of Ophthalmology, Taihe Hospital, Hubei University of Medicine, Shiyan, China

**Keywords:** complex regional pain syndrome, health economic evaluation, pharmacotherapy, interventional management techniques, rehabilitation therapies

## Abstract

**Background:** The economic impact of Complex Regional Pain Syndrome (CRPS) on both patients and the global healthcare system continues to escalate. However, the economic implications associated with management interventions for CRPS have received limited attention. Therefore, our objective is to perform a thorough examination of published economic assessments of the various management strategies utilized for CRPS.

**Methods:** A thorough search spanning four general medical databases and three health economic databases to identify full economic evaluations on CRPS management strategies from January 1994 to June 2023 were conducted. The quality of these studies were evaluated by employing the Consolidated Health Economic Evaluation Reporting Standards (CHEERS) statement. To enable cross-study comparisons conducted in different countries, we adjusted the costs reported in the selected studies for inflation and converted them into 2023 US dollars.

**Results:** A total of nine economic evaluations, consisting of eight high-quality and one medium-quality, were identified across five nations during a span of 29 years. The findings suggest that the most economically efficient intervention for CRPS are interventional approaches of Spinal Cord Stimulation (SCS) in comparison to conventional management for long periods of time. Furthermore, in situations where there is a limited time period of less than 1 year, rehabilitation therapies, particularly physical therapy, have been demonstrated to be more effective in terms of both cost and clinical outcomes.

**Conclusion:** The interventional management strategies, particularly for severe and persistent CRPS over long periods, may offer the greatest cost efficiency. In conditions with limited timelines, rehabilitation measures, such as rehabilitation therapies, can be cost-effective. However, insufficient data for other common interventions prevents the formation of a definitive conclusion. Similarly, it is crucial to recognize that the results of these interventions might be affected by the selection of comparator and the threshold for willingness to pay.

## 1 Introduction

Complex Regional Pain Syndrome (CRPS) is a condition characterized by persistent, spontaneous, or stimulus-evoked chronic pain, frequently impacting a single and exhibiting a duration exceeding 6 months following an initial injury (Marinus et al., 2011). While in the past, different terms have been employed to describe this type of chronic pain (Sebastin, 2011). However, in 1994, in an effort to standardize terminology within the field, the International Association for the Study of Pain (IASP) introduced the term CRPS for it (Merskey, 1994). The condition can be divided into two subcategories based on the absence (CRPS I) or presence (CRPS II) of substantial nerve injury (Petersen et al., 2018).

Regarding etiology, the exact root cause of CPRS remains elusive. Nevertheless, the existing evidence points toward a potential multifaceted source for this issue. Three extensively explored potential possibilities include autonomic dysfunction, neurogenic inflammation, and changes in central nervous system neuroplasticity (Castillo-Guzmán et al., 2015).

The estimated incidence of CPRS ranges from 5.4 to 26.2 per 100,000 person-years (Sandroni et al., 2003; de Mos et al., 2007; Petersen et al., 2018) and the upper limb is more frequently affected, constituting almost 60% of cases (Martínez et al., 2012; Bruehl, 2015). Similarly, there is a higher prevalence among women (with a ratio of 3.4–4 to 1) that occurs between the ages of 46.9–52.7 years (Elsharydah et al., 2017). Furthermore, approximately 40% of cases are associated with fractures or surgical interventions with the primary cause is compression of the median nerve, although instances can also arise after a sprain (10%), root lesions (9%), spinal cord lesions (6%), or even spontaneously (5%–10%) (Martínez et al., 2012).

As the impact of CRPS on both patients and the global healthcare system continues to escalate, there is a growing inclination to comprehend the financial effects of its management approaches (Elsamadicy et al., 2018; Duong et al., 2023). In a study (n = 35,316) by Elsamadicy et al. the costs at the point of CRPS diagnosis, exhibited significant increases in comparison to baseline expenses: the overall costs stood at $8,508; outpatient expenses were $7,251; and pain prescription charges reached $2077. Similarly, over an 8-year period following CRPS diagnosis, the median total cumulative expenditure amounted to $43,026, while pain prescription costs accounted for $12,037 (Elsamadicy et al., 2018). In a separate study, Duong et al. found that healthcare costs were elevated by 20% within the CRPS group compared to the non-CRPS group over a span of 5 years (Duong et al., 2023).

One another important aspect is that the financial strain associated with CRPS goes beyond the direct expenses of medical interventions. In 15%–20% of instances, CRPS develops into a persistent condition, obstructing daily activities and overall life quality. This leads to a 31% incapacity for individuals to return to work within 2 years of symptom onset (Geertzen et al., 1998; de Mos et al., 2007; Bean et al., 2014; van Velzen et al., 2014; Rodham et al., 2016). Due to this reason, patients often contend with considerable losses in productivity, reduced work capacity, and heightened utilization of healthcare resources (Brunner et al., 2008; Marinus et al., 2011; Raja et al., 2021).

The management CRPS often faces delays due to the initial need to rule out other potential sources of pain, such as trauma, neuropathies, and vascular disorders. This comprehensive diagnostic process, while necessary, can extend the time before a definitive diagnosis of CRPS is made and appropriate treatment is initiated (Varenna et al., 2021). Moreover, the management of CRPS necessitates a comprehensive approach that not only targets pain management but also aims to restore functionality to the affected limb (Taylor, 2006; Castillo-Guzmán et al., 2015). This approach encompasses diverse modalities such as pharmacotherapy (Harden et al., 2013), interventional techniques, and rehabilitation therapies (Castillo-Guzmán et al., 2015). The choice of medication in pharmacotherapy depends on the intensity of the pain. For mild pain, traditional NSAIDs like ibuprofen or COX 2 inhibitors such as celecoxib are effective during the acute phase. In cases of more intense pain, controlled release opioids like hydrocodone or oxycodone may be considered.

The interventional techniques are often direct and minimally invasive, aiming to alleviate pain and improve functional outcomes (Ghaly et al., 2023). This includes a variety of procedures, such as sympathetic nerve blocks, which involve the administration of anesthetic injections near sympathetic nerves to decrease pain (Dworkin et al., 2013). Similarly, stellate ganglion blocks target the cervical nerves, effectively easing pain in the upper extremities (Shin and Cheng, 2021). Percutaneous sympathectomy offers a more lasting solution by permanently disrupting sympathetic nerves for prolonged pain relief (Borchers and Gershwin, 2014). In addition, intramuscular injections of Botulinum Toxin A (BTXA) are employed to temporarily paralyze muscles and reduce spasms (Kharkar et al., 2011). Another approach involves the use of cervical or lumbar spinal epidural neurostimulators, which deliver electrical stimulation to the spinal cord to mask pain signals (Martínez et al., 2012). Furthermore, intrathecal pumps, dispensing pain medication directly into the spinal fluid, is another method in this spectrum of interventional techniques (Ghaly et al., 2023).

The rehabilitation therapies (Castillo-Guzmán et al., 2015) encompasses physical, occupational and psychological approaches (Bruehl and Chung, 2006; Taylor et al., 2021). Multiple controlled trials have established physical therapy as a primary line of treatment. This approach initially includes gentle techniques such as elevation, massage, gentle range of motion exercises, and isometric strengthening. As the patient’s condition improves, the intensity of therapy is progressively increased. Additionally, contrast baths, which alternate between heat and cold, and transcutaneous electrical nerve stimulation (TENS) have also been demonstrated to be effective (Sebastin, 2011). In conclusion, given the complexities of CRPS, a multidisciplinary treatment approach is usually recommended. This strategy involves a collaborative effort from healthcare professionals across various disciplines, aiming to comprehensively address the different facets of the condition (Ghaly et al., 2023).

While the health economic assessments play a pivotal role in shaping decision-making processes by evaluating the cost-effectiveness and efficiency of interventions (Khan et al., 2021). However, the cost-effectiveness and economic implications associated with management interventions for CRPS have received limited attention. To bridge this gap, researchers have undertaken diverse health economic evaluations to gauge the financial outcomes linked with varying strategies for managing CRPS. Therefore, our objective is to perform a thorough examination of published economic assessments of the various management strategies utilised for CRPS. By synthesizing the findings from these studies, we aim to provide a comprehensive overview of the economic considerations that accompany various interventions for CRPS, facilitating more informed decision-making and resource allocation in clinical practice.

## 2 Methodology

### 2.1 Search strategy

A comprehensive search of scientific literature, adhering to the guidelines outlined in the Preferred Reporting Items for Systematic Reviews and Meta-Analyses (PRISMA) framework was performed (Moher et al., 2009). The search spanned from January 1994 to June 2023 and the rationale for commencing the data collection from 1994 stems from the fact that the term CRPS was initially introduced by the IASP during that year (Merskey, 1994).

The search encompassed seven databases including PubMed, Google Scholar, Cochrane Central Register of Controlled Trials, Health Technology Assessment (HTA), Cost-Effectiveness Analysis (CEA) registry, the National Health Service Economic Evaluation Database (NHS EED), and ScienceDirect. Furthermore, bibliographic search of relevant journal articles and recent systematic reviews and meta-analyses were also conducted.

To conduct the search, the terms related to “health economics", “CRPS", and “management" were combined. In the PubMed, the keywords for titles/abstracts were formatted as follows:

“Complex Regional Pain Syndrome OR CPRS OR CPRS I OR CPRS II OR Reflex sympathetic dystrophy OR Causalgia OR Sudeck’s atrophy OR Sympathetically mediated pain OR Sympathetically independent pain”

AND

“Treatment strategies OR Therapeutic strategies OR Therapy OR Therapeutics OR Cure OR Management OR Pharmacotherapy OR Pharmacotherapeutics OR Medication OR Neuropathic pain medications OR Anti-inflammatories OR Bisphosphonates OR Antidepressants OR Tricyclic OR Duloxetine OR Venlafaxine OR Gabapentin OR Pregabalin OR opioids OR Tramadol OR Methadone OR Oxycodone OR Codeine OR Buprenorphine OR Morphine OR Toxin Botulinum OR Sympathetic nerve block OR Anti-oxidants OR Vitamin C OR Physical therapy OR Occupational therapy OR Psychological therapy OR Interventional procedures OR Spinal cord stimulation OR SCS OR Dorsal root ganglion OR DRG ”

“Health economics OR Economics OR Economic evaluation OR Economic analysis OR Pharmacoeconomics OR Pharmacoeconomic analysis OR Cost comparison OR Cost analysis OR Cost effectiveness OR Cost-effective analysis OR CEA OR Cost-utility analysis OR Utility analysis OR CUA OR Cost OR Economic cost OR Affordable.”

After removal of duplicates, a thorough scan of titles, abstracts, and full-text articles were conducted to exclude studies that did not meet the inclusion criteria. Reviews, methods or protocol papers, conference papers, case reports, editorials, letters, and correspondences were also screened out during this process. The search strategy for all the databases utilized and the number of obtained results can be found in Supplementary Material 01.

### 2.2 Selection of studies

To streamline the selection criteria for the studies, it was structured as according to the PICOS (Population, Intervention, Comparator, Outcome, Design) framework.

a) Population (P): Individuals who have received a diagnosis of CRPS, aged 18 years or older, without limitations based on gender, severity, or coexisting pathologies.
b) Intervention (I): It includes, though is not confined to, pharmacotherapeutic, interventional, and rehabilitative interventions that have undergone a comprehensive economic analysis comprising both clinical advantages and associated costs.
c) Comparator (C): This could involve comparing the full health economics of different interventions or comparing different intensities or durations of the same intervention.
d) Outcome (O): Encompasses health economic outcomes such as Incremental Cost-Effectiveness Ratio (ICER), cost-benefit ratios, and Incremental Cost-Utility Ratio (ICUR). Furthermore, it includes clinical outcomes pertaining to CRPS management, such as mitigation of pain, enhancement of functional capacity, improvement in quality of life, and the occurrence of adverse events.
e) Study Design (S): It includes randomized controlled trials (RCTs) and observational studies that incorporate health economic evaluations, specifically cost-effectiveness studies, and cost-utility analyses.

In addition to these criteria, studies that solely concentrated on assessing the cost of illness, undertook cost minimization analyses, or conducted partial economic assessments were excluded. Similarly, articles centered around the health financial analysis of screening or diagnostic approaches were not taken into account. Furthermore, studies that only compare various modalities of the same intervention, such as nonrechargeable and rechargeable spinal cord stimulation (SCS) implanted pulse generators, were also not included in the analysis.

### 2.3 Data extraction and quality appraisal

All authors of the study were involved in assessing the study selection process, quality assessment, and data extraction. A standardized data form was used to collect data, which included the first author’s name, the country and year of the study, study design, sample size, gender and age, type of CRPS studied, diagnostic criteria applied, type of intervention and comparator, cost perspective, currency, discount rate, relevant clinical outcomes, associated cost, health economic outcome, limitations, conclusions, and funding information for each study.

To assess the quality of the included economic analyses, the updated Consolidated Health Economic Evaluation Reporting Standards (CHEERS) statement were utilized (Husereau et al., 2022). The updated CHEERS statement can be readily adaptable to various categories of health economic assessments, novel methodologies, and advancements within the discipline. Additionally, it acknowledges the augmented significance of engaging stakeholders, encompassing patients and the general public. The statement holds relevance across a wide spectrum of interventions designed to enhance individual or population health, irrespective of their complexity, and without being bound by specific contexts (Husereau et al., 2022).

The updated CHEERS checklist encompasses 28 components corresponding to distinct sections of economic evaluations. For each question, an answer was made whether it conformed entirely to the stipulated criteria, was not fully met, or was not applicable. A rating was then attributed to each study, calculated based on the proportion of pertinent CHEERS items that were satisfied. Studies achieving 100% compliance with the relevant CHEERS items were classified as demonstrating excellent quality. Those meeting criteria ranging from 75% to 99% were categorized as exhibiting high quality, while those fulfilling criteria from 50% to 74% were deemed to possess moderate quality. Studies that fulfilled less than 50% of the criteria were considered to have low quality.

### 2.4 Data synthesis

The information extracted from the identified economic evaluations was presented through a combination of narrative synthesis and organized tables. The presentation of results adheres to the established guidelines for proficient narrative summaries of health economic investigations, as delineated in the Cochrane Handbook for Systematic Reviews (Shemilt et al., 2008).

To enable cross-study comparisons conducted in different countries, all costs were recalibrated from their initial currencies and price years and then converted into US dollars ($) for the 2023 price year, employing the methods established by the Campbell and Cochrane Economics Methods Group Evidence for Policy and Practice Information and Coordinating Centre (EPPI-centre, 2019). The specific information regarding the original costs and their conversion to US dollars ($) for the year 2023 can be found in the Supplementary Material 02.

## 3 Results

### 3.1 Literature search

**FIGURE 1:**
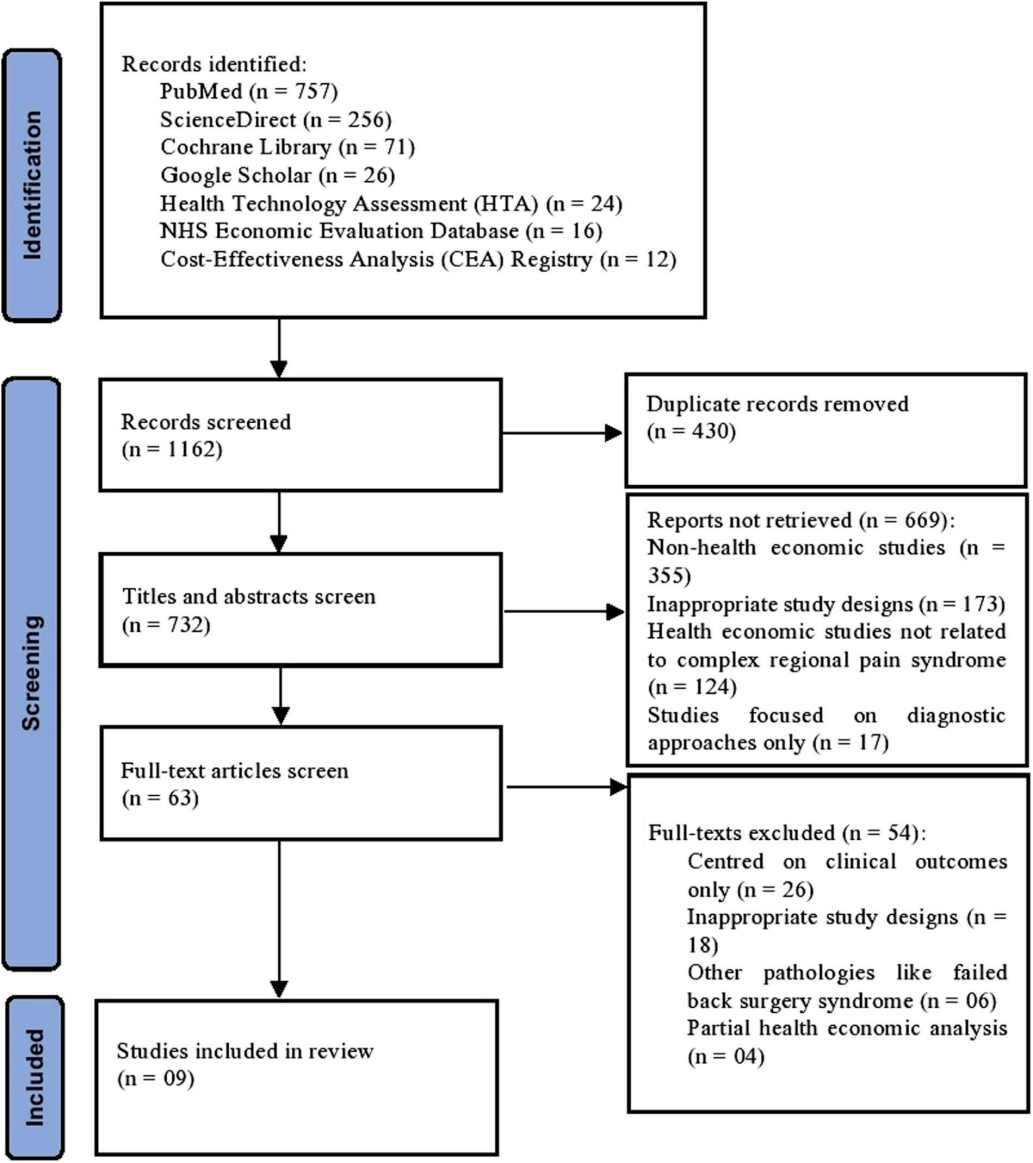
Prisma. Flow chart of the searching and screening studies.

A comprehensive search of seven databases initially yielded a total of 1,162 records. Following the removal of 430 duplicates, 732 studies underwent an initial abstract and title screening. Among these, 669 studies were excluded based on predetermined criteria outlined in Figure 1. Subsequently, during a thorough full-text review of the remaining 63 articles, 54 studies were further excluded for reasons such as an exclusive focus on clinical outcomes, inappropriate study designs, addressing other pathologies or were partial health economic analysis. In the end, nine studies (Barnhoorn et al., 2018; den Hollander et al., 2018; Kemler and Furnée, 2002; Kemler et al., 2010; Kumar and Rizvi, 2013; Mekhail et al., 2018; Severens et al., 1999; van Dieten et al., 2003; Zinboonyahgoon et al., 2023) spanning from January 1994 to June 2023 were selected for inclusion in the analysis, that focused on comparing the clinical and cost outcomes associated with various management strategies for CRPS (Figure 1).

### 3.2 Quality assessment of included studies

According to the assessment criteria against the updated CHEERS statement (Husereau et al., 2022), none of the studies selected provided comprehensive information covering all the specified elements. Out of the chosen studies, one received a medium-quality rating (Barnhoorn et al., 2018), while the remaining eight received high-quality ratings (den Hollander et al., 2018; Kemler and Furnée, 2002; Kemler et al., 2010; Kumar and Rizvi, 2013; Mekhail et al., 2018; Severens et al., 1999; van Dieten et al., 2003; Zinboonyahgoon et al., 2023) as shown in Table 1.

**TABLE 1:** Quality of health economic studies for the management strategies of complex regional pain syndrome (CRPS) against Consolidated Health Economic Evaluation Reporting Standards (CHEERS) statement.

Study	CHEERS items satisfied	CHEERS items not satisfied	Relevant CHEERS items	Percent (%) satisfied	Quality
Zinboonyahgoon et al. (2023)	23	5	28	82	High
Mekhail et al. (2018)	23	5	28	82	High
Barnhoorn et al. (2018)	18	7	25	72	Medium
den Hollander et al. (2018)	20	5	25	80	High
Kumar and Rizvi (2013)	23	5	28	82	High
Kemler et al. (2010)	22	6	28	79	High
van Dieten et al. (2003)	21	4	25	84	High
Kemler and Furnée (2002)	19	6	25	76	High
Severens et al. (1999)	19	6	25	76	High

^a^ The comprehensive evaluation of the quality of included studies against CHEERS, statement is provided in the supplementary file 03.

With the introduction of new elements in the updated CHEERS statement, several areas were insufficiently addressed in the majority of the studies. For instance, only one study (Barnhoorn et al., 2018) included details concerning health economic analysis plans within their methods section. Similarly, the characterization of distributional effects within the methods section, as well as the approach to engaging with patients and other stakeholders affected by the study in both the methods and results sections, were absent in all the studies. A comprehensive evaluation of the selected studies' adherence to the CHEERS statement can be found in the Supplementary Material 03.

### 3.3 Baseline characteristics

Among the selected studies, the majority, totaling five, were conducted in the Netherlands (Barnhoorn et al., 2018; den Hollander et al., 2018; Kemler and Furnée, 2002; Severens et al., 1999; van Dieten et al., 2003) while the remaining four took place in Thailand (Zinboonyahgoon et al., 2023), the USA (Mekhail et al., 2018), Canada (Kumar and Rizvi, 2013), and the UK (Kemler et al., 2010). There were four studies that followed a modeling approach (Kemler et al., 2010; Kumar and Rizvi, 2013; Mekhail et al., 2018; Zinboonyahgoon et al., 2023), and the remaining five studies were based on trials (Barnhoorn et al., 2018; den Hollander et al., 2018; Kemler and Furnée, 2002; Severens et al., 1999; van Dieten et al., 2003), the follow up period for all studies ranges from 6 months to 20 years.

The review encompassed a total of 684 participants, with an average age of 48.2 years, and approximately 63% of the participants were female. The focus of the majority of these studies was on CRPS I (Barnhoorn et al., 2018; den Hollander et al., 2018; Kemler and Furnée, 2002; Kemler et al., 2010; Severens et al., 1999; van Dieten et al., 2003), with only one study, presented the findings pertaining to both CRPS-I and CRPS-II (Mekhail et al., 2018). There were two studies that provided data on chronic pain, but we specifically focused on extracting data related to CRPS only (Kumar and Rizvi, 2013; Zinboonyahgoon et al., 2023). Regarding the diagnostic criteria employed across these studies for CRPS-I, three studies applied the IASP criteria (Merskey, 1986), while two studies each utilized the Veldman criteria (Veldman et al., 1993) and the Budapest criteria (Harden et al., 2007). The baseline characteristics of included studies is presented in Table 2.

**TABLE 2:** Methodological characteristics of health economic studies on complex regional pain syndrome (CRPS) management strategies.

Study	Country	Study type	CRPS type	Diagnostic criteria	Sample	Intervention	Comparator	Primary clinical outcome	Perspective	Time horizon	Discount rate
Zinboonyahgoon et al. (2023)	Thailand	Model based prospective observational study	NR	NR	n = 03; age 47.4 years; 45% women	SCS + CMM	CMM	QALY using the EQ-5D (Thai version)	Societal	03 years	3%
Mekhail et al. (2018)	United States	Model based retrospective cost-utility analysis	CRPS I and CRPS II	Budapest criteria for CRPS I	n = 152; age 52.4 years; 51.3% women	DRG stimulation	SCS	QALY using the SF-36	U.S. payer perspective	10 years	3%
Barnhoorn et al. (2018)	Netherlands	RCT	CRPS I	Budapest criteria	n = 56; age 44.3 years; 80.4% women	Pain exposure PT	CMM	QALY using the EQ-5D	Healthcare	09 months	NA
den Hollander et al. (2018)	Netherlands	RCT	CRPS I	IASP criteria	n = 46; age 44.4 years; 84.2% women	Exposure *in vivo* (CBT)	Pain-contingent PT	QALY using the SF-36	Societal	06 months	NA
Kumar and Rizvi (2013)	Canada	Model based prospective observational study	NR	NR	n = 53; age 50.5 years; 46% women	SCS + CMM	CMM	QALY using the EQ-5D	Canadian provincial ministry of health	20 years	3.5%
Kemler et al. (2010)	United Kingdom	Model based on RCT	CRPS-I	IASP criteria	n = 54 years; age 18–65 years	SCS + CMM	CMM	QALY using the EQ-5D	United Kingdom National Health Services	15 years	3.5%
van Dieten et al. (2003)	Netherlands	Double-dummy, double-blind, RCT	CRPS I	Veldman criteria	n = 131; age 50 years; 63.5% women	Acetylcysteine	DMSO	QALY using the EQ-5D	Societal	01 years	NA
Kemler and Furnée (2002)	Netherlands	RCT	CRPS I	IASP criteria	n = 54; age 18–65 years	SCS + PT	PT	QALY using the EQ-5D	Societal	01 years and extended till expected death	3%
Severens et al. (1999)	Netherlands	RCT	CRPS I	Veldman criteria	n = 135; 70% women	PT	OT	ISS, Greentest, and SIP score	Societal	01 years	NA

CBT, cognitive behavioral therapy; CMM, comprehensive medical management; CT, control treatment; CRPS, complex regional pain syndrome; DMSO, dimethylsulfoxide; DRG, dorsal root ganglion; ED-5D, EuroQol-5, dimensions; ISS, Impairment-level Sum Score; IASP, international association for the study of pain; NA, not applicable; OT, occupational therapy; PT, physical therapy; QALY, Quality-Adjusted Life Year; RCT, randomized controlled trial; SF-36, Short Form 36; SIP, sickness impact profile.

### 3.4 Reported costs

Out of the nine studies, three conducted health economics evaluations using both cost-effective and cost-utility analyses (van Dieten et al., 2003; Mekhail et al., 2018; Zinboonyahgoon et al., 2023). The remaining six studies solely focused on cost-effectiveness analysis (Barnhoorn et al., 2018; den Hollander et al., 2018; Kemler and Furnée, 2002; Kemler et al., 2010; Kumar and Rizvi, 2013; Severens et al., 1999). In all of these studies, various components of direct medical costs were taken into account, encompassing intervention cost, hospitalization, outpatient services, diagnostic/lab services, and medication. However, noteworthy is that five of these studies (Barnhoorn et al., 2018; den Hollander et al., 2018; Severens et al., 1999; van Dieten et al., 2003; Zinboonyahgoon et al., 2023) additionally included data related to direct non-medical costs, which included out-of-pocket expenses and transportation costs. Furthermore, only three studies (den Hollander et al., 2018; Severens et al., 1999; van Dieten et al., 2003) reported indirect costs related to productivity losses. Specifically, all studies reported intervention costs, followed by medication costs in 78% of the studies. The least frequently reported cost category was out-of-pocket expenses, covered in only 22% of the studies. In terms of the analytical perspective, majority of the studies, i.e., 5/9 adopted a societal viewpoint in their analyses (den Hollander et al., 2018; Kemler and Furnée, 2002; Severens et al., 1999; van Dieten et al., 2003; Zinboonyahgoon et al., 2023). Moreover, for studies with follow-up periods exceeding 1 year, discount rates applied ranged from 3% to 3.5%. The breakdown of costs included in the studies is represented in Table 3.

**TABLE 3:** Breakdown of costs included in the health economic studies for the management strategies of complex regional pain syndrome (CRPS).

Type of cost	Zinboonyahgoon et al. (2023)	Mekhail et al. (2018)	Barnhoorn et al. (2018)	den Hollander et al. (2018)	Kumar and Rizvi (2013)	Kemler et al. (2010)	van Dieten et al. (2003)	Kemler and Furnée (2002)	Severens et al. (1999)	Proportion of studies (%)
Direct medical cost										
Intervention	+	+	+	+	+	+	+	+	+	100
Hospitalization	+	+		+	+				+	56
Outpatient services	+			+	+		+			33
Diagnostic/Lab services	+	+			+					33
Medication	+	+	+	+	+		+		+	78
Direct non-medical cost										
Out of pocket expenses				+					+	22
Transportation	+		+	+			+		+	56
Indirect cost										
Productivity losses				+			+		+	33

### 3.5 Reported health outcomes

In eight out of the nine studies, the overall health status was evaluated using Quality-Adjusted Life Years (QALY) as a standardized measurement (Barnhoorn et al., 2018; den Hollander et al., 2018; Kemler and Furnée, 2002; Kemler et al., 2010; Kumar and Rizvi, 2013; Mekhail et al., 2018; van Dieten et al., 2003; Zinboonyahgoon et al., 2023). The EQ-5D questionnaire was commonly utilized as the assessment tool for collecting this QALY data. Notably, one study conducted by Severens et al. exclusively focused on clinical outcomes, employing metrics such as the Impairment-level Sum Scale (ISS), Greentest, and the Sickness Impact Profile (SIP) (Severens et al., 1999). Moreover, besides the QALY measurements, three of the studies incorporated clinical outcome data. One study (Barnhoorn et al., 2018) utilized the ISS and the pain disability index to gauge pain-related disability. Similarly, Van Dieten et al. employed the ISS score to evaluate impairment (van Dieten et al., 2003). Lastly, pain levels were quantified using the Visual Analog Scale (VAS) score in one study (Kemler and Furnée, 2002) (Table 2).

### 3.6 Interventional characteristics of included studies

There were diverse types of interventions that were examined in the selected studies. These interventions can be broadly grouped into three categories as outlined below.

#### 3.6.1 Pharmacotherapy

Only one study (n = 131) examined the pharmacotherapeutic interventions over a timeframe of 1 year (van Dieten et al., 2003). It involved administering 600 mg acetylcysteine tablets thrice daily and applying 50% (Dimethylsulfoxide) DMSO cream five times a day. Patients with CRPS in the upper extremities received occupational therapy, and those with CRPS in the lower extremities received physical therapy (PT). Moreover, patients were evaluated at the start and at 6, 17, 32, and 52 weeks after treatment initiation. Both intervention groups demonstrated relevant ISS improvements, but the differences in ISS and utility between them did not reach statistical significance. Subgroup analysis indicated that DMSO appeared more effective in treating warm CRPS, whereas the results were reversed for cold CRPS. In warm CRPS, DMSO significantly improved ISS and utility over the 0–17 week period compared to acetylcysteine, with significant cost differences over the 0–52 week period. Therefore, from both clinical and economic perspectives, DMSO cream was concluded as generally preferable to acetylcysteine for most warm CRPS patients.

#### 3.6.2 Interventional techniques

The interventional techniques were extensively examined in our selected set of five studies. The predominant technique employed was SCS (Kemler and Furnée, 2002; Kemler et al., 2010; Kumar and Rizvi, 2013; Mekhail et al., 2018; Zinboonyahgoon et al., 2023). Among these studies, SCS was compared to comprehensive medical management (CMM) in three of them (Kemler et al., 2010; Kumar and Rizvi, 2013; Mekhail et al., 2018; Zinboonyahgoon et al., 2023) while in the remaining one study, it was compared against PT (Kemler and Furnée, 2002). Remarkably, in each of these studies, SCS implantation was carried out exclusively after achieving a 50% reduction in pain intensity from the baseline during the trial period. Furthermore, the selected studies investigated both rechargeable and non-rechargeable implantable pulse generators (IPG), except for a single study (Kemler and Furnée, 2002). Similarly, in only two studies, the details of stimulation parameters (pulse width and amplitude) were reported (Kemler and Furnée, 2002; Mekhail et al., 2018).

In four out of five studies (n = 313), it was determined that SCS was a cost-effective option for extended timeframes, ranging from 10 years to a lifetime (Kemler and Furnée, 2002; Kemler et al., 2010; Kumar and Rizvi, 2013; Mekhail et al., 2018). Notably, SCS in combination with CMM was regarded as cost-effective in three of these studies (Kemler et al., 2010; Kumar and Rizvi, 2013; Mekhail et al., 2018), compared to CMM alone. In the fourth study (Kemler and Furnée, 2002), both SCS and PT were found to be more cost-effective than PT alone. Furthermore, in two of these investigations, there was a comparison between rechargeable and non-rechargeable IPGs. These studies concluded that when the lifespan of an IPG is limited to either 4 years (Kemler et al., 2010) or 4.25 years (Kumar and Rizvi, 2013), or even less, a rechargeable IPG, despite its initial higher cost, emerges as a more economically efficient choice compared to a non-rechargeable IPG. In addition to the comparison with CMM, a study also conducted a comparative analysis between SCS and dorsal root ganglion (DRG) stimulation. In that study, despite the higher costs associated with DRG, primarily due to permanent implantation, it emerged as the more advantageous therapy, leading to an improved quality of life when compared to SCS (Mekhail et al., 2018).

There was a study found that combining SCS with CMM was not deemed cost-effective when compared to CMM alone over a period of 3 years in Thailand, regardless of whether rechargeable or non-rechargeable IPGs were used. This particular study involved a cohort of more than 29 patients suffering from chronic refractory pain, with only three of them having CRPS. The conclusion was primarily driven by the values of ICUR that exceeded the established willingness-to-pay (WTP) threshold of 160,000 THB/QALY gained in Thailand (Zinboonyahgoon et al., 2023).

#### 3.6.3 Rehabilitation therapies

There were three studies (n = 237) that concluded rehabilitation therapies as cost effective over a period of 6 months to 1 year. Two of these studies, found that PT was cost effective then occupational therapy and CMM (Severens et al., 1999; Barnhoorn et al., 2018). In the third study, it was observed that exposure *in vivo* was cost effective then then pain-contingent PT (den Hollander et al., 2018).

In the study of Barnhoorn et al. a specific treatment approach of PT known as pain exposure physical therapy (PEPT) was examined. PEPT consisted of a maximum of five treatment sessions, each lasting 40 min, with the intervals between sessions adjusted based on the patient’s progress and individual needs. The clinical effectiveness of PEPT was assessed at multiple time points, including baseline, 3 months, 6 months, and 9 months. While, there were no significant differences in terms of QALYs (mean difference = −0.02; 95% confidence interval (CI) −0.10 to 0.04) and clinical outcomes between the two groups. Nevertheless, a cost minimization analysis revealed that PEPT, when compared to CMM, was considered cost-effective (Barnhoorn et al., 2018).

In the second study, there was an absence of comprehensive details about the PT protocol. The participants underwent assessments at four different time points: 6 weeks, 3 months, 6 months, and 12 months. Notably, there was a significant positive difference in the case of the ISS when comparing PT with occupational therapy and CMM. Moreover, the ICER for PT *versus* occupational therapy and CMM were moderate or even indicated dominance, demonstrating that PT was both more effective and more cost-efficient than the comparison treatments (Severens et al., 1999).

Lastly, in a study (n = 46), exposure *in vivo* was compared to pain-contingent PT. Exposure *in vivo* is a specific technique commonly employed within the realm of cognitive-behavioral therapy (CBT). The results of the study suggest that exposure *in vivo* led to more significant improvements in QALY. Despite the initial higher treatment costs associated with exposure *in vivo*, there was an observable trend toward reducing overall expenses, especially in the realm of healthcare costs. As a result, exposure *in vivo* emerges as a more cost-effective option when compared to pain-contingent PT (den Hollander et al., 2018). The summary of the interventional characteristics in health economic studies for CRPS is presented in Table 4.

**TABLE 4:** Summary of interventional characteristics in health economic studies for complex regional pain syndrome (CRPS).

Study	Intervention 01			Intervention 02			ICER/ICUR	WTP threshold	Conclusion
	Type	Net cost	Net outcome	Type	Net cost	Net outcome			
Zinboonyahgoon et al. (2023)	SCS + CMM; both rechargeable and non-rechargeable for ≥50% pain reduction in trial stimulation	$187,945	10.34 QALY	CMM	$71,396	8.22 QALY	$54,834/QALY gained	$13,706/QALY gained	SCS + CMM was not a cost-effective strategy
Mekhail et al. (2018)	DRG stimulation; pulse width, 301.8us and amplitude 832.4uA	$174,001	4.96 QALY	SCS; pulse width, 422.6us and amplitude 3278.3uA	$144,935	4.58 QALY	$76,943/QALY gained	<$112993/QALY = cost-effective, and <$56,497/QALY = highly cost-effective	DRG and SCS were cost-effective when compared to CMM alone, and DRG was more cost-effective than SCS
Barnhoorn et al. (2018)	PEPT; maximum of five physical therapy sessions	$2,398	0.76 QALY	CMM	$3,466	0.74 QALY	PEPT dominant		PEPT cost effective
den Hollander et al. (2018)	Exposure *in vivo* (CBT); three session of 1 h each	$40,975	0.62 QALY	Pain-contingent PT; rest, tissue massage, TENS, and pain-reducing exercises	$62,194	0.55 QALY	Exposure *in vivo* (CBT) dominant	$23,047	Exposure *in vivo* (CBT) cost effective
								/QALY gained and $115,235	
								/QALY gained	
Kumar and Rizvi (2013)	SCS + CMM; both rechargeable and non-rechargeable for ≥50% pain reduction in trial stimulation	$167,627	4.24 QALY	CMM	$144,531	2.12 QALY	$10,894/QALY gained	$48,566/QALY gained	SCS + CMM cost-effective
Kemler et al. (2010)	SCS + CMM; both rechargeable and non-rechargeable types	$162,365	4.84 QALY	CMM	$149,276	2.88 QALY	$6,665/QALY gained	$37,424/QALY gained to $56,136/QALY gained	SCS + CMM cost-effective
van Dieten et al. (2003)	Acetylcysteine; 600mg, taken three times daily for 52 weeks	$9,912	0.62 QALY	DMSO; 50% cream applied five times daily for 52 weeks	$7,222	0.64 QALY	$-21,316/QALY gained		DMSO as the preferred treatment for warm CRPS
Kemler and Furnée (2002)	SCS + PT; pulse width, 210usec and amplitude 0–10V, for ≥50% pain reduction in trial stimulation	$11,943,679	0.43 QALY	PT; 30 min twice a week for 6 months	$8,012,014	0.22 QALY	SCS + PT dominant		SCS + PT cost effective for chronic CRPS
Severens et al. (1999)	PT	$17,654	15.8 (ISS score)	OT	$26,450	14.2 (ISS score)	PT dominant		PT cost effective

^a^ All costs from their initial currency and price year have been updated into U.S., dollars (USD) for the 2023 price year utilizing the cost converter tool of Campbell and Cochrane Economics Methods Group Evidence for Policy and Practice Information and Coordinating Centre. The specific information regarding the original costs and their conversion to USD, for the year 2023 can be found in the Supplementary Material 02.

^b^ ICER/ICUR, values may not be reproducible because of variations in rounding on reported net cost and outcome.

CBT, cognitive behavioral therapy; CMM, conventional medical management; DRG, dorsal root ganglion; ICER, Incremental Cost-Effectiveness Ratio; ICUR, Incremental Cost-Utility Ratio; ISS, Impairment-level Sum Score; OT, occupational therapy; PEPT, pain exposure physical therapy; PT, physical therapy; QALY, Quality-Adjusted Life Year; SCS, spinal cord stimulation; WTP, willingness to pay.

## 4 Discussion

Based on the comprehensive systematic review that encompassed nine separate studies investigating the cost-effectiveness of various management strategies for CRPS, the overall consensus leaned towards favoring interventional approaches for extended timeframes, severe and refractory CRPS, with a notable emphasis on SCS. Additionally, in cases with a restricted time frame of less than 1 year, particularly rehabilitation therapies, were found to be cost-effective. The conclusion is supported by the observation that studies which deemed intervention techniques as cost-effective employed extended timeframes. Conversely, there was a single study with a relatively shorter follow-up of 3 years, and led to the opposite conclusion for interventional strategy of SCS. Similarly, the reverse situation applies to studies that considered non-interventional approaches as cost-effective. However, it is crucial to acknowledge that the study outcomes were influenced by factors such as the comparison treatments used, and the willingness-to-pay criteria. Similarly, it is worth noting that a substantial majority (67%) of the selected studies were conducted in the Netherlands, indicating a notable geographical concentration, and were predominantly centered on interventional strategies.

To the best of our knowledge, this is the first systematic review that aimed to bridge existing gaps in the literature by consolidating and analyzing health economic evaluations related to CRPS management strategies. There was a prior review by Taylor et al., in 2006 but that did delve into SCS only for CRPS. Similarly, it encompassed both clinical and cost-effectiveness studies. Furthermore, it is noteworthy that this previous review incorporated only a single comprehensive economic analysis (Taylor, 2006). While in our case, the studies typically centered around CRPS I, which can be attributed to its higher prevalence when compared to CRPS II (Misidou and Papagoras, 2019). However, since there is no distinction in the management strategies for CRPS (Bruehl and Chung, 2006; Harden et al., 2013; Castillo-Guzmán et al., 2015), the findings can be applied broadly to both types.

In total of 9 selected studies, 8 were deemed to be of good quality based on the updated CHEERS statement (Husereau et al., 2022). The previous CHEERS statement comprised 24 elements (Husereau et al., 2013), however the revised statement incorporated an additional 4 components, yielding a total of 28. In our case, only one study specifically addresses the newly added item of health economic analysis plans (Barnhoorn et al., 2018), while none of the studies adequately address the other three newly added items.

While pharmacotherapy is usually considered as the primary treatment approach for CRPS, the analysis reveals that only one study has explored this aspect (van Dieten et al., 2003). According to the findings, DMSO emerged as the favored treatment for warm CRPS when compared to acetylcysteine. CRPS is characterized by two distinct phases: the acute (warm) phase and the chronic (cold) phase (Misidou and Papagoras, 2019). Consequently, it can be deduced that DMSO generally demonstrates greater effectiveness in addressing the acute stages of the syndrome. Moreover, the disparities between warm and cold RSD are believed to be linked to differences in blood circulation. Nevertheless, there is a notable absence of experimental data to ascertain whether these distinctions affect the absorption of DMSO cream (Yu and Quinn, 1994). It is important to note that while some studies have also supported the clinical effectiveness of DMSO but these studies were based on older literature (Geertzen et al., 1994; Zuurmond et al., 1996; Perez et al., 2003). Therefore, there is a need to investigate the cost-effectiveness of alternative drugs that are commonly used in current medical practice (Mangnus et al., 2022). Moreover, a more robust conclusion can be reached by comparing the use of pharmacotherapy with an alternative management strategy, such as interventional or rehabilitation treatment.

In five of the studies, interventional techniques were incorporated, with four of them reaching the conclusion that these management strategies were cost-effective. The inclination towards focusing on these strategies in majority of the studies can be attributed to the ease of evaluating both cost and clinical benefits associated with interventions like SCS and DRG stimulation. Three of these studies expressed a preference for SCS (Kemler and Furnée, 2002; Kemler et al., 2010; Kumar and Rizvi, 2013), while one endorsed DRG stimulation (Mekhail et al., 2018).

A similar trend of conclusions, particularly favoring SCS, has been consistently observed in previous literature, particularly in the context of similar conditions like failed back surgery syndrome (Taylor, 2006; Niyomsri et al., 2020; McClure et al., 2021). However, it is essential to consider various factors when applying these findings in clinical practice. Across all five of these studies, interventional approaches were primarily compared against CMM. However, the details regarding these CMM showed variation (O'Connell et al., 2013) and the selected studies did not provide comprehensive information about these CMM, except for one study that explicitly mentioned PT (Kemler and Furnée, 2002). Furthermore, it would lead to more conclusive results if the examined technique were compared to a variety of distinct interventions. Only one study, ventured into evaluating the cost-effectiveness of SCS and DRG stimulation (Mekhail et al., 2018). Similarly, it is worth noting that the studies that concluded interventional strategies as cost effective were conducted in the USA (Mekhail et al., 2018), Canada (Kumar and Rizvi, 2013), and two in the Netherlands (Kemler and Furnée, 2002; Kemler et al., 2010). It is crucial to acknowledge that the willingness-to-pay threshold can vary significantly from one country to another (Woods et al., 2016). Therefore, when interpreting the results, it is essential to consider the specific context of the country in which the study was conducted.

The single study that determined that SCS + CMM was not a cost-effective strategy when compared to CMM alone had a relatively small sample size (n = 3) for CRPS. Consequently, the results from this study may be considered insignificant. Additionally, the conclusion was based on a WTP threshold specific to Thailand, which may not be applicable or generalizable to other studies or settings (Zinboonyahgoon et al., 2023).

The studies focusing on rehabilitation therapies displayed a wide range of interventions. However, one particularly noteworthy intervention is PEPT, that is specifically designed for individuals with CRPS. PEPT involves a progressive-loading exercise program and addresses the management of pain-avoidance behavior (Ek et al., 2009; van de Meent et al., 2011). While there is existing data regarding the clinical effectiveness of this innovative technique (Staal et al., 2019), it is advisable to undergo more extensive cost-effectiveness research for PEPT. Relying solely on a single included study may not suffice for drawing robust conclusions (Barnhoorn et al., 2018). Similarly, the same principle applies to exposure *in vivo*, a technique whose clinical effectiveness has been examined in studies related to chronic pain (Goossens et al., 2015; Chowdhury et al., 2022). However, further in-depth research is warranted, particularly in the context of CRPS.

In addition to the interventions examined in the review, there were many interventions often used in routine practice that were not evaluated by any specific study. For example, sympathetic nerve block is typically regarded as the primary interventional therapy option for patients with CRPS (Wie et al., 2021). However, study that assess the cost-effectiveness of this interventions was not found. The same applies to the health economic analysis of other techniques, such as BTXA. Finally, the review only examined two drugs, neither of which are commonly used in everyday clinical settings (Charlton, 2005; Sebastin, 2011). We did not find any studies that assessed the cost-effectiveness of medications currently utilized in clinical practice. These reasons make it extremely difficult to reach a robust and all-encompassing judgement.

This review is subject to certain limitations. All the studies included in the analysis were carried out exclusively in specific countries: Thailand, the United States, the United Kingdom, Canada, and the Netherlands and we did not found any economic evidence relevant to low and middle-income countries. It is crucial to acknowledge that the findings may not be universally applicable to nations with diverse economic contexts, given the variations in economic systems across countries. Furthermore, while there was consistency in assessing health-related benefits, the studies differed in their approach to evaluating costs, encompassing direct medical costs, direct non-medical costs, and indirect costs. Additionally, adverse effects were only reported in a single study (Mekhail et al., 2018). This variability made it challenging to draw meaningful comparisons across these different categories.

This review has pinpointed several areas that warrant attention in future research endeavors. Firstly, there is a need for more comprehensive economic analyses that employ larger and more representative sample sizes. Moreover, economic analyses should be conducted in other countries, with a particular focus on low and middle-income nations, in order to broaden the applicability of the findings. The focus should be on interventions that have received less attention in previous research, such as pharmacotherapy and sympathetic nerve block. Furthermore, it is of utmost importance to compare treatment strategies that employ diverse approaches, given that the majority of the studies reviewed exclusively compared interventions with CMM. This comparative methodology is indispensable for achieving more definitive conclusions concerning the various strategies applied in clinical practice.

## 5 Conclusion

The interventional approaches and SCS in particular, have the potential to be highly beneficial in terms of both cost and outcomes for long-term, severe CRPS. Similarly, rehabilitation interventions such as physical therapy, might offer cost-efficient solutions in cases with restricted timeframes. Nevertheless, the lack of enough data for other commonly used management strategies like pharmacotherapy hinders the establishment of a conclusive statement. It is important to understand that the outcomes of these treatments can be influenced by the choice of comparator and the threshold for willingness to pay.

The review has brought to light several critical areas warranting attention in future research. Firstly, there is an urgent demand for more comprehensive economic analyses that incorporate larger and more representative sample sizes. Furthermore, it is crucial to carry out economic evaluations in various nations, especially those with low and middle-income statuses, and on unexplored interventions, in order to enhance the applicability of the findings.

## Data Availability

The original contributions presented in the study are included in the article/Supplementary Material, further inquiries can be directed to the corresponding author.
