# Supplementary material for: Economic evaluation of management strategies for complex regional pain syndrome (CRPS)
Source: Front Pharmacol. 2024 Jan 22;15:1297927. doi: 10.3389/fphar.2024.1297927 (PMC10839065; doi:10.3389/fphar.2024.1297927)
Supplement: Supplementary file 1 [file Table1.DOCX]

Table. Search strategy for the included databases

| **Data Base** | **Search Terms** | **Results** |
| --- | --- | --- |
| PubMed | ((Cost) AND (Spinal cord stimulation)) AND (complex regional pain syndrome (CRPS)) | 22 |
| PubMed | ((Cost) AND (Psychological therapy)) AND (complex regional pain syndrome (CRPS)) | 06 |
| PubMed | ((Cost) AND (Occuptional therapy)) AND (complex regional pain syndrome (CRPS)) - Spellcheck off | 00 |
| PubMed | ((Cost) AND (occupational therapy)) AND (complex regional pain syndrome (CRPS)) | 01 |
| PubMed | ((Cost) AND (Physical therapy)) AND (complex regional pain syndrome (CRPS)) | 24 |
| PubMed | ((Cost) AND (Rehibilitation)) AND (complex regional pain syndrome (CRPS)) - Spellcheck off | 00 |
| PubMed | ((Cost) AND (rehabilitation)) AND (complex regional pain syndrome (CRPS)) | 24 |
| PubMed | ((Cost) AND (Surgery)) AND (complex regional pain syndrome (CRPS)) | 20 |
| PubMed | ((Cost) AND (Pharmacotherapy)) AND (complex regional pain syndrome (CRPS)) | 11 |
| PubMed | ((Cost-utility analysis) AND (Interventions)) AND (complex regional pain syndrome (CRPS)) | 09 |
| PubMed | ((Cost effectiveness) AND (Interventions)) AND (complex regional pain syndrome (CRPS)) | 18 |
| PubMed | ((Cost effectiveness) AND (treatment)) AND (complex regional pain syndrome (CRPS)) | 20 |
| PubMed | ((Cost comparison) AND (treatment)) AND (complex regional pain syndrome (CRPS)) | 16 |
| PubMed | ((Health economics) AND (treatment)) AND (complex regional pain syndrome (CRPS)) | 37 |
| PubMed | ((Complex regional pain syndrome OR CPRS OR CPRS I OR CPRS II OR Reflex sympathetic dystrophy OR Causalgia OR Sudeck’s atrophy OR Sympathetically mediated pain OR Sympathetically independent pain) AND (Treatment strategies OR Therapeutic strategies OR Therapy OR Therapeutics OR Cure OR Management OR Pharmacotherapy OR Pharmacotherapeutics OR Medication OR Neuropathic pain medications OR Anti-inflammatories OR Bisphosphonates OR Sympathetic nerve block OR Anti-oxidants OR Vitamin C OR Botulinum toxin OR Physical therapy OR Occupational therapy OR Psychological therapy OR Interventional procedures OR Surgery OR Spinal cord stimulation OR SCS OR Dorsal root ganglion OR DRG)) AND (Health economics OR Economics OR Economic evaluation OR Economic analysis OR Pharmacoeconomics OR Pharmacoeconomic analysis OR Cost comparison OR Cost analysis OR Cost effectiveness OR Cost-effective analysis OR CEA OR Cost-utility analysis OR Utility analysis OR CUA OR Cost OR Economic cost OR Affordable) | 550 |
| ScienceDirect | "health economics" "treatment" and "complex regional pain syndrome (CRPS)" | 49 |
| ScienceDirect | "Cost effectiveness" AND "Interventions" AND "complex regional pain syndrome (CRPS)" | 207 |
| Cochrane Library | "Cost" AND "treatment" AND "complex regional pain syndrome" | 30 trials |
| Cochrane Library | "Cost effectiveness" AND "treatment" AND "complex regional pain syndrome" | 18 trials |
| Cochrane Library | "Cost effectiveness" AND "Interventions" AND "complex regional pain syndrome" | 05 trials |
| Cochrane Library | "Cost effectiveness" AND "pharmacotherapy" AND "complex regional pain syndrome" | 00 trials |
| Cochrane Library | "Cost effectiveness" AND "Surgery" AND "complex regional pain syndrome" | 06 trials |
| Cochrane Library | "Cost effectiveness" AND "Rehabilitation" AND "complex regional pain syndrome" | 06 trials |
| Cochrane Library | "Cost effectiveness" AND "Psychological therapy" AND "complex regional pain syndrome" | 00 trials |
| Cochrane Library | "Cost effectiveness" AND "Spinal cord stimulation" AND "complex regional pain syndrome" | 06 trials |
| Cochrane Library | ("health economics" AND "treatment" AND "complex regional pain syndrome (CRPS)"):ti,ab,kw" (Word variations have been searched) | 00 trials |
| Google Scholar | allintitle: health economics AND treatment AND Complex regional pain syndrome | 00 |
| Google Scholar | allintitle: Cost AND treatment AND Complex regional pain syndrome | 04 |
| Google Scholar | allintitle: Cost utility AND Interventions AND Complex regional pain syndrome | 02 |
| Google Scholar | allintitle: Cost effectiveness AND Interventions AND Complex regional pain syndrome | 03 |
| Google Scholar | Cost effectiveness AND Interventions AND Complex regional pain syndrome | 05 (Selected on the basis of titles from first 10 articles) |
| Google Scholar | health economics AND Interventions AND Complex regional pain syndrome | 03 (Selected on the basis of titles from first 10 articles) |
| Google Scholar | health economics AND pharmacotherapy AND Complex regional pain syndrome | 04 (Selected on the basis of titles from first 10 articles) |
| Google Scholar | health economics AND Surgery AND Complex regional pain syndrome | 03 (Selected on the basis of titles from first 10 articles) |
| Google Scholar | health economics AND Physical therapy AND Complex regional pain syndrome | 02 (Selected on the basis of titles from first 10 articles) |
| NHS EED | Health economics AND Complex regional pain syndrome AND Treatment | 00 |
| NHS EED | Cost AND Complex regional pain syndrome AND Treatment | 07 |
| NHS EED | Cost AND Complex regional pain syndrome AND Management | 04 |
| NHS EED | (Cost effectiveness AND Complex regional pain syndrome AND Pharmacotherapy) FROM 1994 TO 2023 | 00 |
| NHS EED | (Cost effectiveness AND Complex regional pain syndrome AND Surgery) FROM 1994 TO 2023 | 05 |
| HTA | "Cost" AND "treatment" AND "complex regional pain syndrome (CRPS)" | 01 |
| HTA | Cost AND Management AND complex regional pain syndrome | 01 |
| HTA | Cost AND complex regional pain syndrome | 02 |
| HTA | complex regional pain syndrome | 12 |
| HTA | Pharmacotherapy AND complex regional pain syndrome | 00 |
| HTA | Rehabilitation AND complex regional pain syndrome | 02 |
| HTA | Spinal cord stimulation AND complex regional pain syndrome | 06 |
| CEA Registry | complex regional pain syndrome | 05 |
| CEA Registry | Management AND complex regional pain syndrome | 03 |
| CEA Registry | Pharmacotherapy AND complex regional pain syndrome | 00 |
| CEA Registry | Surgery AND complex regional pain syndrome | 01 |
| CEA Registry | Rehabilitation AND complex regional pain syndrome | 00 |
| CEA Registry | Spinal cord stimulation AND complex regional pain syndrome | 03 |
