# Supplementary material for: Economic evaluation of management strategies for complex regional pain syndrome (CRPS)
Source: Front Pharmacol. 2024 Jan 22;15:1297927. doi: 10.3389/fphar.2024.1297927 (PMC10839065; doi:10.3389/fphar.2024.1297927)
Supplement: Supplementary file 2 [file Table2.DOCX]

Table. Costs Associated with Interventions and Corresponding Year of Costing

| **Study** | **Net Cost (Exp.)** | **Net Cost (Control)** | **Cost year** | **Economic outcome** |
| --- | --- | --- | --- | --- |
| Zinboonyahgoon et al., 2023 | THB2,194,001.16 | THB833,451.09 | 2021 | THB640,113.8/QALY gained |
| Mekhail et al., 2021 | $153,992 | $128,269 | 2017 | $68,095/QALY gained |
| Barnhoorn et al., 2018 | €1648 | €2382 | 2013 |  |
| den Hollander et al., 2018 | €28,446 | €43,177.29 | 2015 |  |
| Kumar & Rizvi, 2013 | CAN$172,577 | CAN$148,799 | 2013 | CAN$11,216/QALY gained |
| Kemler et al., 2010 | £86,770 | £79,775 | 2008 | £3562/QALY gained |
| van Dieten et al., 2003 | €5580 | €4066 | 2001 | €-12000/QALY gained |
| Kemler & Furnée, 2002 | €6161507 | €4,133,239 | 1998 |  |
| Severens et al., 1999 | €8692 | €13,023 | 1996 |  |

Table. Costs Associated with Interventions and in USD Year 2023

| **Study** | **Net Cost (Exp.)** | **Net Cost (Control)** | **Economic outcome** |
| --- | --- | --- | --- |
| Zinboonyahgoon et al., 2023 | $187,944.73 | $71,395.93 | $54,834.07/QALY gained |
| Mekhail et al., 2021 | $174,000.63 | $144,935.37 | $76,942.78/QALY gained |
| Barnhoorn et al., 2018 | $2,398.25 | $3,466.40 |  |
| den Hollander et al., 2018 | $40,974.51 | $62,193.93 |  |
| Kumar & Rizvi, 2013 | $167,626.93 | $144,530.96 | $10,894.29/QALY gained |
| Kemler et al., 2010 | $162,364.63 | $149,275.54 | $6665.24  /QALY gained |
| van Dieten et al., 2003 | $9,911.71 | $7,222.41 | $-21,315.52/QALY gained |
| Kemler & Furnée, 2002 | $11,943,679.23 | $8,012,014.07 |  |
| Severens et al., 1999 | $17,653.52 | $26,449.82 |  |
