# Supplementary material for: Economic evaluation of management strategies for complex regional pain syndrome (CRPS)
Source: Front Pharmacol. 2024 Jan 22;15:1297927. doi: 10.3389/fphar.2024.1297927 (PMC10839065; doi:10.3389/fphar.2024.1297927)
Supplement: Supplementary file 3 [file Table3.DOCX]

Table. Quality assessment of included studies by using CHEERS checklist

| **Topic** | **No.** | **Item** | **Zinboonyahgoon et al., 2023** | **Mekhail et al., 2021** | **Barnhoorn et al., 2018** | **den Hollander et al., 2018** | **Kumar & Rizvi, 2013** | **Kemler et al., 2010** | **van Dieten et al., 2003** | **Kemler & Furnée, 2002** | **Severens et al., 1999** |
| --- | --- | --- | --- | --- | --- | --- | --- | --- | --- | --- | --- |
| **Title** | 01 | Identify the study as an economic evaluation and specify the interventions being compared. | Y | Y | Y | Y | Y | Y | Y | Y | Y |
| **Abstract** | 02 | Provide a structured summary that highlights context, key methods, results, and alternative analyses. | Y | Y | Y | Y | Y | Y | Y | Y | Y |
| **INTRODUCTION** | | | | | | | | | | | |
| **Background and objectives** | 03 | Give the context for the study, the study question, and its practical relevance for decision making in policy or practice | Y | Y | Y | Y | Y | Y | Y | Y | Y |
| **METHODS** | | | | | | | | | | | |
| **Health economic analysis plan** | 04 | Indicate whether a health economic analysis plan was developed and where available | N | N | Y | N | N | N | N | N | N |
| **Study population** | 05 | Describe characteristics of the study population (such as age range, demographics, socioeconomic, or clinical characteristics) | Y | Y | Y | Y | Y | Y | Y | Y | Y |
| **Setting and location** | 06 | Provide relevant contextual information that may influence findings. | Y | Y | Y | Y | Y | Y | Y | Y | N |
| **Comparators** | 07 | Describe the interventions or strategies being compared and why chosen. | Y | Y | Y | Y | Y | Y | Y | Y | Y |
| **Perspective** | 08 | State the perspective(s) adopted by the study and why chosen. | Y | Y | N | Y | Y | Y | Y | Y | N |
| **Time horizon** | 09 | State the time horizon for the study and why appropriate. | Y | Y | Y | Y | Y | Y | Y | Y | Y |
| **Discount rate** | 10 | Report the discount rate(s) and reason chosen. | Y | Y | NA | NA | Y | Y | NA | NA | NA |
| **Selection of outcomes** | 11 | Describe what outcomes were used as the measure(s) of benefit(s) and harm(s) | Y | Y | Y | Y | Y | Y | Y | Y | Y |
| **Measurement of outcomes** | 12 | Describe how outcomes used to capture benefit(s) and harm(s) were measured | Y | Y | Y | Y | Y | Y | Y | Y | Y |
| **Valuation of outcomes** | 13 | Describe the population and methods used to measure and value outcomes | Y | Y | Y | Y | Y | Y | Y | Y | Y |
| **Measurement and valuation of resources and costs** | 14 | Describe how costs were valued. | Y | Y | Y | Y | Y | Y | Y | Y | Y |
| **Currency, price date, and conversion** | 15 | Report the dates of the estimated resource quantities and unit costs, plus the currency and year of conversion. | Y | Y | Y | Y | Y | Y | Y | Y | Y |
| **Rationale and description of mode** | 16 | If modelling is used, describe in detail, and why used. Report if the model is publicly available and where it can be accessed. | Y | Y | NA | NA | Y | Y | NA | NA | NA |
| **Analytics and assumptions** | 17 | Describe any methods for analysing or statistically transforming data, any extrapolation methods, and approaches for validating any model used | Y | Y | NA | NA | Y | Y | NA | NA | NA |
| **Characterising heterogeneity** | 18 | Describe any methods used for estimating how the results of the study vary for subgroups. | N | N | N | N | N | N | Y | Y | Y |
| **Characterising distributional effects** | 19 | Describe how impacts are distributed across different individuals or adjustments made to reflect priority populations. | N | N | N | N | N | N | N | N | N |
| **Characterising uncertainty** | 20 | Describe methods to characterise any sources of uncertainty in the analysis. | Y | Y | N | Y | Y | Y | Y | Y | Y |
| **Approach to engagement with patients and others affected by the study** | 21 | Describe any approaches to engage patients or service recipients, the general public, communities, or stakeholders (such as clinicians or payers) in the design of the study. | N | N | N | N | N | N | N | N | N |
| **RESULTS** | | | | | | | | | | | |
| **Study parameters** | 22 | Report all analytic inputs (such as values, ranges, references) including uncertainty or distributional assumptions. | Y | Y | Y | Y | Y | Y | Y | Y | Y |
| **Summary of main results** | 23 | Report the mean values for the main categories of costs and outcomes of interest and summarise them in the most appropriate overall measure. | Y | Y | Y | Y | Y | Y | Y | Y | Y |
| **Effect of uncertainty** | 24 | Describe how uncertainty about analytic judgments, inputs, or projections affect findings. Report the effect of choice of discount rate and time horizon, if applicable. | Y | Y | N | Y | Y | Y | Y | Y | Y |
| **Effect of engagement with patients and others affected by the study** | 25 | Report on any difference patient/service recipient, general public, community, or stakeholder involvement made to the approach or findings of the study | N | N | N | N | N | N | N | N | N |
| **DISCUSSION** | | | | | | | | | | | |
| **Study findings, limitations, generalisability, and current knowledge** | 26 | Report on any difference patient/service recipient, general public, community, or stakeholder involvement made to the approach or findings of the study | Y | Y | Y | Y | Y | Y | Y | Y | Y |
| **OTHER RELEVANT INFORMATION** | | | | | | | | | | | |
| **Source of funding** | 27 | Describe how the study was funded and any role of the funder in the identification, design, conduct, and reporting of the analysis | Y | Y | Y | Y | Y | Y | Y | N | Y |
| **Conflict of interest** | 28 | Report authors conflicts of interest according to journal or International Committee of Medical Journal Editors requirements. | Y | Y | Y | Y | Y | N | Y | N | Y |
